# Epidemic Changes and Spatio-Temporal Analysis of Japanese Encephalitis in Shaanxi Province, China, 2005–2018

**DOI:** 10.3389/fpubh.2020.00380

**Published:** 2020-08-07

**Authors:** Shuxuan Song, Hongwu Yao, Zurong Yang, Zhen He, Zhongjun Shao, Kun Liu

**Affiliations:** ^1^Department of Epidemiology, Ministry of Education Key Lab of Hazard Assessment and Control in Special Operational Environment, School of Public Health, Air Force Medical University, Xi'an, China; ^2^The First Medical Center, Chinese PLA General Hospital, Beijing, China; ^3^Centre for Disease Prevent and Control in Northern Theater Command, Shenyang, China

**Keywords:** Japanese encephalitis, epidemic changes, environmental factors, spatio-temporal analysis, panel data

## Abstract

Japanese encephalitis (JE) is a mosquito-borne viral disease, which is the most serious viral encephalitis in China and other countries of the Asia-Pacific region. Since 2005, the epidemic patterns of JE have changed dramatically in China because of the vaccination of children younger than 15 years old, and JE is expanding geographically along with global warming. This retrospective epidemiological study analyzed dynamic environmental factors and the spatio-temporal distribution of human cases of JE in Shaanxi Province—one of the most severely affected areas of China—from 2005 to 2018. The results demonstrated that the high-risk population changed rapidly as the annual rate of JE cases increased by more than 40% in the age group >60 years during the study period, and endemic areas expanded northward in Shaanxi. Hotspot analysis detected four hotspots accounting for 52.38% the total cases, and the panel negative binomial regression model revealed that the spatio-temporal distribution of JE was significantly affected by temperature, relative humidity, wind velocity, El Niño-Southern Oscillation, coniferous forest coverage, and urban areas. These findings can provide useful information for improving current strategies and measures to reduce disease incidence.

## Introduction

Japanese encephalitis (JE) is a mosquito-borne viral disease caused by the Japanese encephalitis virus (JEV), which belongs to the *Flaviviridae* family and the genus *Flavivirus* (1–3). The disease is clinically characterized by fever, headache, nausea and vomiting, lowered level of consciousness, seizures, movement disorders, and acute flaccid paralysis, and ~30–50% of the patients who survive may experience severe neurological and mental sequelae (4–6). JEV is transmitted predominantly by the mosquito species *Culex tritaeniorhynchus* (*Cx. tritaeniorhynchus*), which is the primary vector and reproduces in stagnant water, including irrigated rice paddies, groundwater, and marshes (5, 7). Pigs and ardeid birds are the amplifying hosts (8), whereas humans are the dead-end hosts. JE is mainly distributed in Asia and Northern Australia, and the estimated annual number of cases and deaths worldwide is ~68,000 and 10,000–15,000, respectively (7, 9). China accounts for 50% of the reported cases worldwide (9) and has categorized JE as a class B notifiable infectious disease since 1951 (10). More than 2 million cases of JE were reported between 1950 and 2011 (11) in China, representing a considerable health care burden. With the beginning of a national immunization program in the early 1970s, JE incidence decreased dramatically, and most cases were sporadic. However, adult cases and outbreaks have occurred more frequently in recent years, indicating that the epidemiological characteristics of JE are changing (12).

Shaanxi Province is an administrative province in Northwestern China. In August 1951, the first suspected cases were reported in Xi'an city (the capital of Shaanxi) and were confirmed as epidemic encephalitis B by serology and viral isolation; since then, this disease has been reported in various parts of Shaanxi Province (13). Shaanxi's position in the national ranking for the incidence of JE moved from 18th in the 1960s to second in the 1990s (13). The incidence of JE has decreased gradually in Shaanxi since the implementation of an immunization program for children in 2005. However, the number of JE cases has increased in recent years.

As a mosquito-borne infectious disease, previous studies have shown that the epidemics of JE were associated with ecological factors (host animals, geographical landscape, topography, and climate), and anthropogenic factors (agricultural activities and urbanization) (14–16). Appropriate weather conditions are necessary for the survival, reproduction, distribution, and spread of disease pathogens, vectors, and hosts, and human exposure to JE is usually determined by the environmental and seasonal distribution of *Cx. tritaeniorhynchus*. A key research priority for preventing and controlling JE is improving the basic understanding of the epidemiological features and the factors underlying disease transmission (15, 17). Therefore, this retrospective epidemiological study used geographic information system (GIS) spatial analyses, multivariable ecological models, and long-term surveillance data from Shaanxi Province from 2005 to 2018 to delineate dynamic epidemic change patterns and high-risk areas of JE in Shaanxi and identify the environmental factors associated with the spatio-temporal distribution of the disease.

## Materials and Methods

### Study Area

Shaanxi Province is an administrative province in Northwestern China located between 31°42' to 39°35' north latitude and 105°29' to 111°15' east longitude. The total area is 2,058,000 km2 with a population of 38,131,293 in 2017. Shaanxi Province is divided into three subregions according to climate and landforms: Shaannan mountainous area, Guanzhong plain, and Shaanbei plateau (18).

### Data Collection and Management

In China, JE is categorized as a class B notifiable infectious disease, and cases diagnosed at medical institutions are required to be reported to the local Center for Disease Control and Prevention (CDC) through the National Notifiable Infectious Diseases Reporting Information System within 12 h in urban areas or 24 h in rural areas. A confirmed JE case was defined according to the guidelines of the World Health Organization, as detailed in a previous study (19). In this study, data on human cases of JE from January 1, 2005 to December 31, 2018 were obtained from the CDC of Shaanxi Province, including sex, age, occupation, geographical location, date of onset of symptoms, date of hospital admission, and clinical outcomes, and all records were anonymized before analysis. Demographic data for each county were obtained from the Shaanxi Statistical Yearbook from 2005 to 2018.

According to the published studies and expert opinions, the factors potentially associated with the transmission of JE were determined in our study. Weather data in China, including temperature, relative humidity, wind velocity, and El Niño-Southern Oscillation (ENSO) were obtained from the Meteorological Data Sharing Service System. Elevation data were collected from the China Meteorological Data Service Center (available at http://data.cma.cn/). Land cover data were derived from a raster version of the “GlobCover land cover map,” which was processed by the European Space Agency (20). Land cover types were classified as follows: cropland, herbaceous cover, transition between cropland and natural vegetation, broad-leaved forest coverage, coniferous forest coverage, shrub or herbaceous coverage, shrubland, grassland, sparse vegetation, water bodies, bare land, and urban areas. Data on pig density were collected from the Food and Agriculture Organization of the United Nations.

### Statistical Analysis

#### Epidemiological Characteristics

Data on annual incidences were plotted to display the seasonal distribution of JE using the “ggridges” package in R software version 3.6.2 to visualize changes in distribution over time. The annual incidence of JE in each county of Shaanxi Province was georeferenced on a digital map to illustrate the spatial-temporal distribution of JE. The number of cases and the incidence of JE over the study period were stratified by sex and age group to evaluate demographic characteristics.

#### Analysis of Spatio-Temporal Hotspots

To identify the areas most affected by JE, circular spatial scan statistic was computed using SaTScan software version 9.4 (21). A discrete Poisson-based model was constructed to detect disease hotspots, defining the maximum spatial size as ≤ 15% of the total population and the maximum temporal size as ≤ 85% of the study period. This algorithm is used to evaluate the random distribution or spatial and temporal clustering of diseases and test statistical significance as well as to monitor diseases and their geographic/spatial determinants and detect outbreaks prospectively (22, 23). The statistical significance of hotspots was determined by Monte Carlo hypothesis testing, which simulated 999 random replications under the null hypothesis to ensure adequate power to identify significant high-risk areas. A *p*-value of <0.05 was considered significant. Epidemiological and ecological features were described for each hotspot, and the location of hotspots were shown on an incidence map.

#### Panel Negative Binomial Regression

A panel negative binomial regression model was used to assess the potential environmental risk factors for the occurrence of JE at the county level from 2005 to 2018. Panel data are used to analyze cross-sectional and time-series data, which increased the degrees of freedom and reduced collinearity among explanatory variables (24). Negative binomial regression is used to model count data, with the condition that data variance is much higher than its mean (25), and it is adequate for over-dispersed count data. In this model, the annual incidence of JE from 2005 to 2018 was defined for each county as the outcome, and 19 potential factors as independent variables. Multivariable analysis (26) was conducted using the selected variables with a *p*-value of <0.05 in the univariable analysis after determining the collinearity among the variables. The incidence rate ratio (IRR) in response to changes in each variable was calculated to assess the impact of each variable, and a *p*-value of <0.05 in the multivariable model was considered statistically significant. All analyses were performed using STATA software version 16.0 (StataCorp LP, College Station TX, USA).

#### Ethical Statement

The National Health Commission of China determined whether ethical approval was required for this study. In China, the collection of data from human Japanese encephalitis cases was part of routine public health surveillance, and such data collection was exempt from institutional review board assessment.

## Results

**Figure 1:**
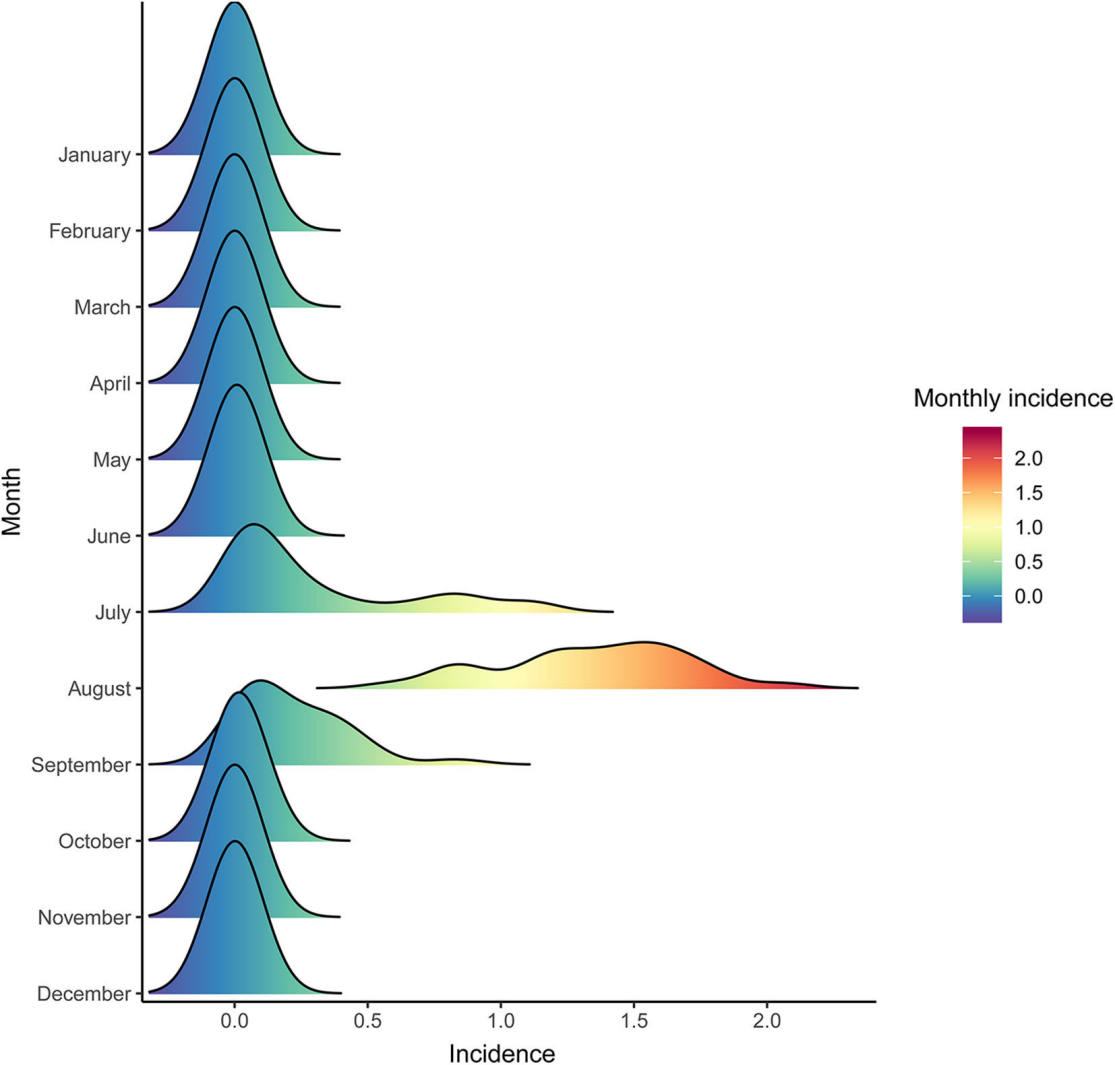
Average monthly incidence of Japanese encephalitis in Shaanxi Province, China, 2005–2018.

**Figure 2:**
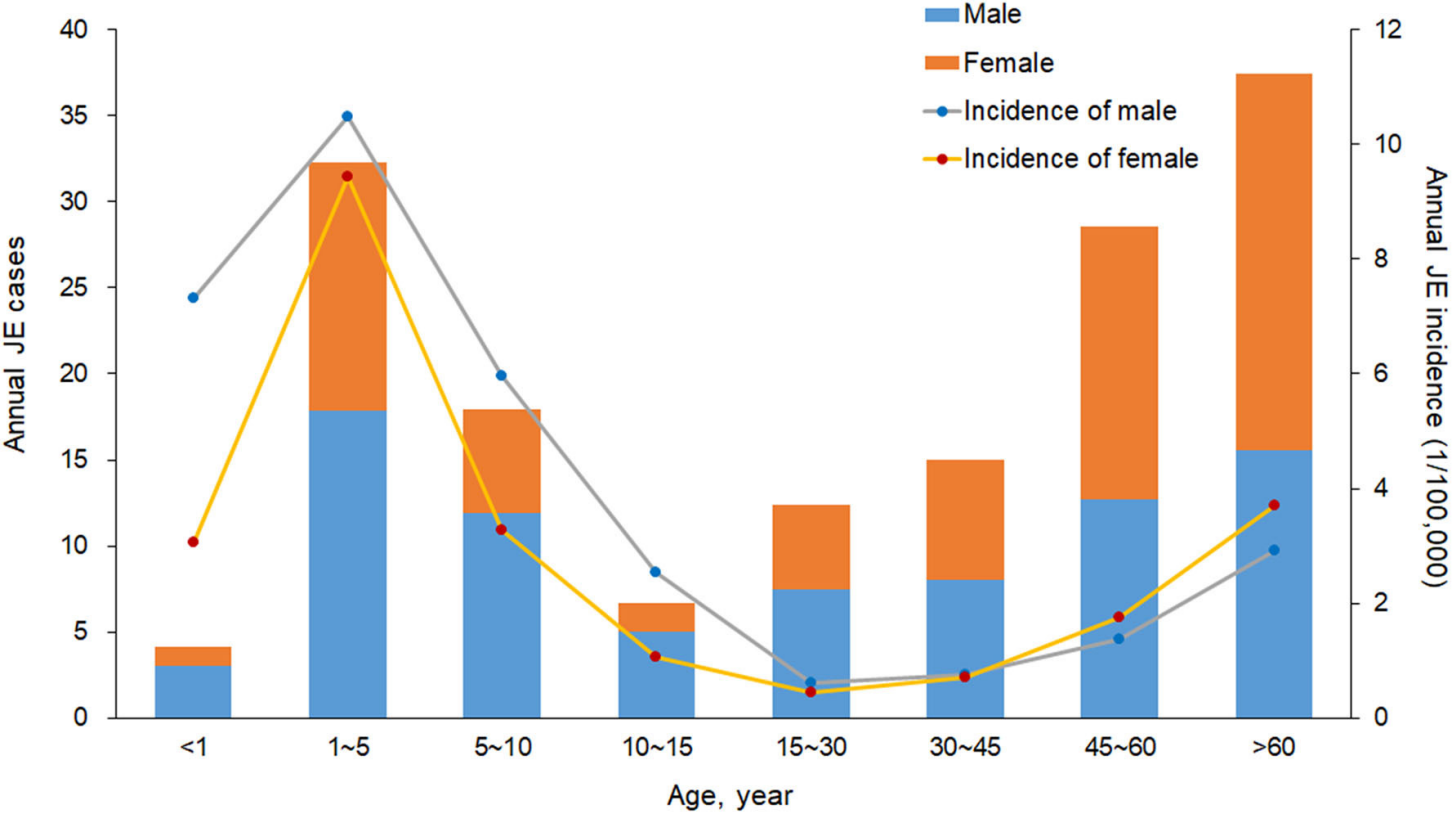
Annual number of cases and incidence (1/100,000) of Japanese encephalitis by sex and age group in Shaanxi Province, China, 2005–2018.

**Figure 3:**
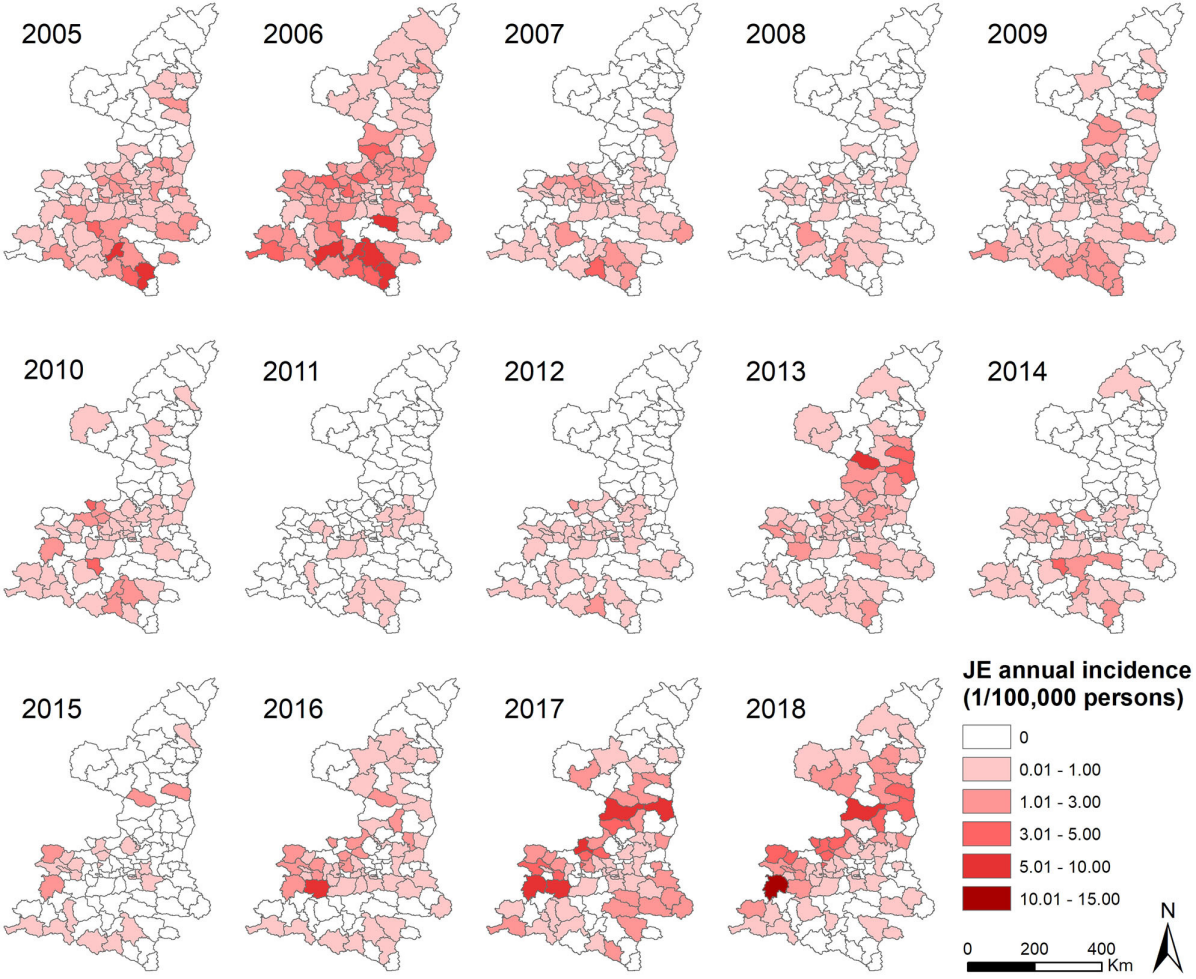
Annual incidence of Japanese encephalitis per county in Shaanxi Province, China, 2005–2018.

A total of 2,161 confirmed cases and 81 deaths due to JE were reported in Shaanxi from 2005 to 2018. The results revealed a seasonal peak, with 98.3% cases occurring from June to September each year, and the number of cases was highest in August (Figure 1). The annual incidence varied from 0.08 cases per 100,000 persons in 2011 to 1.38 cases per 100,000 persons in 2006. The male-to-female ratio of all cases was 1.12, and the median age of the affected population was 35.5 years (range, 0 to 95) (Table 1). The number of cases was significantly higher in the age groups 1–5 years and >60 years (Figure 2). Moreover, the number of cases in the age group >60 years increased by more than 40% in the study period. All 107 counties in Shaanxi reported human cases of JE, and the annual incidence varied from 0 to 14.32 cases per 100,000 persons. The five counties with the highest annual incidence were Changwu, Feng, Hanyin, Taibai, and Pingli, with 31.03, 29.39, 22.79, 21.60, and 20.73 cases per 100,000 persons, respectively. The spatio-temporal analysis showed that the disease expanded northward (Figure 3).

**Table 1:** Distribution of patients with Japanese encephalitis according to sex, age group, and profession in Shaanxi Province, China, 2005–2018.

**Year**	**Total cases**	**Sex ratio (Male:female)**	**Age group (years)**			**Occupation**					
			**<15 (%)**	**15–60 (%)**	**>60 (%)**	**Peasants (%)**	**Students (%)**	**Scattered children (%)**	**Child-care children (%)**	**Retirees (%)**	**Others (%)**
2005	213	1.13	65.26	25.35	9.39	28.64	22.07	27.30	4.78	1.88	40.85
2006	514	1.20	52.53	33.27	14.20	35.41	18.48	24.30	3.98	2.72	38.13
2007	123	1.05	56.10	30.08	13.82	35.77	22.76	22.64	3.77	0.00	36.59
2008	46	1.42	58.70	28.26	13.04	32.61	26.09	24.59	3.28	2.17	34.78
2009	169	1.32	50.89	37.87	11.24	34.32	15.98	24.22	3.59	4.14	40.83
2010	101	1.06	56.44	29.70	13.86	30.69	19.80	27.86	2.14	2.97	43.56
2011	28	1.80	64.29	17.86	17.86	21.43	32.14	26.32	0.00	7.14	39.29
2012	64	1.21	46.88	34.38	18.75	45.31	14.06	23.81	1.19	4.69	34.38
2013	154	0.75	21.43	42.21	36.36	66.88	7.79	11.49	0.57	4.55	20.13
2014	58	1.32	46.55	37.93	15.52	39.66	24.14	19.44	1.39	5.17	29.31
2015	40	1.35	32.50	42.50	25.00	47.50	32.50	9.09	0.00	2.50	17.50
2016	119	0.89	12.61	45.38	42.02	71.43	10.08	4.03	0.81	5.88	11.76
2017	310	1.46	15.16	46.13	38.71	68.39	8.71	5.78	2.13	2.58	18.06
2018	222	0.75	3.15	45.95	50.90	82.88	5.41	0.00	0.00	4.05	7.66

**Figure 4:**
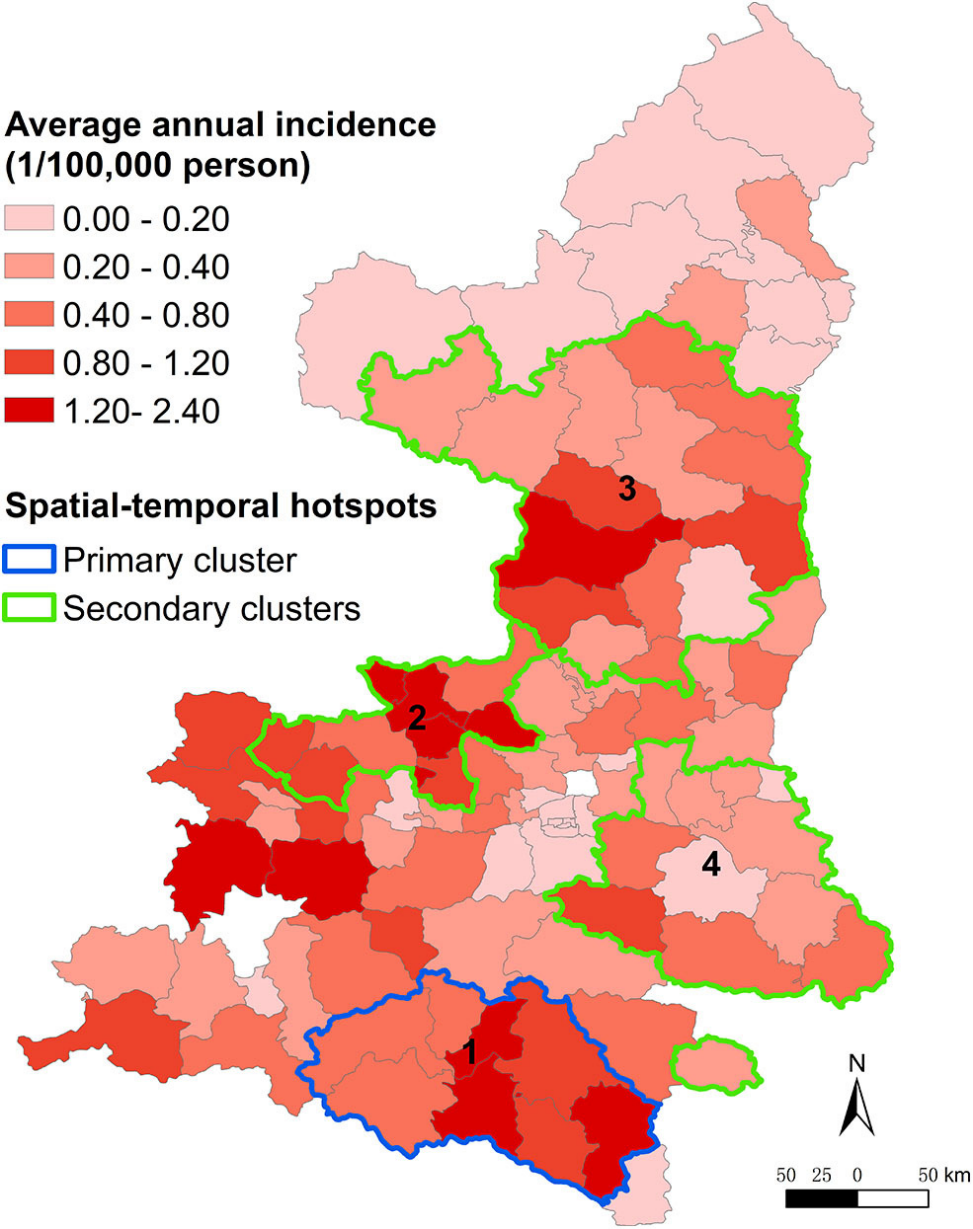
Spatio-temporal hotspots representing areas in Shaanxi Province, China, with different incidences of Japanese encephalitis from 2005 to 2018.

Spatio-temporal scan statistics identified four significant disease hotspots covering 43 counties, which accounted for 52.38% of the total cases (Figure 4). The primary hotspot (hotspot 1) included eight counties and the period from January 2005 to December 2006, with a risk ratio (RR) of 9.86. Hotspot 2 covered nine counties and the period from January 2016 to December 2018, with a RR of 6.11. Hotspot 3 included 15 counties with a RR of 5.03. Hotspot 4 comprised 12 counties and the period from January 2005 to December 2006, with a RR of 2.66 (Table 2). Hotspot 1 was located in southern Shaanxi, hotspots 2 and 4 were situated in the central region, and hotspot 3 was located in the northern region.

**Table 2:** Information for spatio-temporal hotspots of Japanese encephalitis in Shaanxi Province, China, 2005–2018.

**Hotspots**	**Hotspot 1**	**Hotspot 2**	**Hotspot 3**	**Hotspot 4**
Time period	2005/1/1–2006/12/31	2016/1/1–2018/12/31	2017/1/1–2018/12/31	2005/1/1–2006/12/31
No. obs	190	167	104	98
No. exp	20.95	29.25	21.53	37.97
RR	9.86	6.11	5.03	2.66
LLR	256.852529	157.81014	82.94617	33.76037
Annual incidence	3.7	2.3	2.0	1.1
No. counties	8	9	15	12
No. Population	2,545,548	2,434,685	2,576,281	4,494,775
Area (Km^2^)	19,297.63	10,334.48	39,428.5	23,042.28
Major geomorphology	Plain	Plain	Hills and Loess Plateau	Mountainous area
Major land cover	Tree cover and irrigated cropland	Cropland	Rainfall cropland	Tree cover and irrigated cropland
Elevation, median (range)	973 m (759–1263)	1099 m (742–1379)	1199 m (847–1432)	956 m (505–1354)

*No. obs, number of observed cases; No. exp, number of expected cases; RR, relative risk for the JE incidence in the hotspots compared to the average incidence at the same time period; LLR, log likelihood ratio; No. counties, number of counties within hotspot; No. population, population within the hotspot*.

The risk factors associated with annual spatio-temporal variations in JE were determined by panel negative binomial regression. In the univariable analysis, 11 factors—temperature, relative humidity, wind speed, ENSO, human population density, pig density, broad-leaved forest coverage, coniferous forest coverage, shrub or herbaceous coverage, urban areas, and water bodies—were significantly associated with the annual incidence of JE. The final multivariable model indicated that six factors played significant roles in the spatio-temporal distribution of JE in Shaanxi, including four risk factors—temperature (adjusted hazard ratio [HR], 1.50; 95% confidence interval [CI], 1.39–1.61; *P* < 0.001), relative humidity (adjusted HR, 1.05; 95% CI, 1.02–1.08; *P* < 0.001), wind velocity (adjusted HR, 1.39; 95% CI, 1.32–1.47; *P* < 0.001), and ENSO (adjusted HR, 1.27; 95% CI, 1.18–1.36; *P* < 0.001); and two protective factors—coniferous forest coverage (adjusted HR, 0.95; 95% CI, 0.91–0.98; *P* = 0.003), and urban areas (adjusted HR, 0.98; 95% CI, 0.98–0.99; *P* < 0.001) (Table 3).

**Table 3:** Association between the annual incidence of Japanese encephalitis and potential risk factors by panel negative binomial regression.

**Variables**	**Univariable analysis**		**Multivariable analysis**	
	**Crude IRR (95% CI)**	***P***	**Adjusted IRR (95% CI)**	***P***
Pig density	0.99 (0.99–1.00)	0.016	NS (excluded)	
Human population density	0.99 (0.99–1.00)	<0.001	NS (excluded)	
Temperature	1.08 (1.02–1.14)	0.005	1.50 (1.39–1.61)	<0.001
Relative humidity	1.02 (1.01–1.04)	0.002	1.05 (1.02–1.08)	<0.001
Wind velocity	1.10 (1.05–1.15)	<0.001	1.39 (1.32–1.47)	<0.001
El Niño-Southern Oscillation	1.07 (1.01–1.14)	0.024	1.27 (1.18–1.36)	<0.001
Broad-leaved forest coverage	1.01 (1.00–1.01)	0.067	NS (excluded)	
Shrub or herbaceous coverage	0.85 (0.74–0.99)	0.032	NS (excluded)	
Water bodies coverage	0.76 (0.56–1.02)	0.072	NS (excluded)	
Coniferous forest coverage	1.03 (1.00–1.06)	0.080	0.95 (0.91–0.98)	0.003
Urban areas coverage	0.98 (0.98–0.99)	<0.001	0.98 (0.98–0.99)	<0.001

## Discussion

JEV is the most important causative agent of viral encephalitis in China, with 13,773 cases reported from 2010 to 2018 (27). The present findings indicated that (i) the age group with the highest risk of JE shifted in Shaanxi, such that the number of cases decreased gradually in the age group <15 years and increased significantly in the age group >60 years; (ii) spatio-temporal cluster analysis detected four hotspots accounting for 52.38% of the total reported cases during the study period, and the disease spread northward significantly; (iii) panel negative binomial regression analysis revealed that the spatio-temporal distribution of JE was significantly correlated with temperature, relative humidity, wind velocity, ENSO, coniferous forest coverage, and urban areas.

Most cases (75%) occurred in children aged <15 years (9). However, there were significant changes in the epidemiological characteristics of JE, such that the number of cases increased by more than 40% in the population older than 60 years and decreased by more than 60% in the population younger than 15 years. This shift might be related to two factors. The first is the national immunization program for children younger than 15 years ^1^. JE vaccine was integrated in the China National Expanded Program on Immunization (EPI) integrated all provinces at the end of 2007, while it was integrated in the Childhood Immunization Programme of Shaanxi Province at 2004, and two doses of the vaccine SA14-14-2 are required in Shaanxi Province: the first dose at the age of 8 months, and the second (booster) dose at the age of 2 years (28). Thanks to the implement of vaccination, the cases of JE decreased dramatically from 0.33–5.40/100,000 during 1978 to 2008, to 0.12-0.29/100,000 since 2008 (11). The second factor is related to the migration of young adults from rural areas to cities to find better jobs, leaving the elderly in charge of agricultural activities in villages, increasing exposure to mosquitoes, which are the primary hosts, and this phenomenon may explain the rapid increment in the number of peasants. Therefore, the risk of JE among elderly farmers in rural areas increased, highlighting the need to improve vaccination coverage in the age group >60 years in highly endemic areas.

The results indicated that JE spread slowly northward over the study period. Most cases occurred in the southern region of Shaanxi after an outbreak of JE in 2006. However, the incidence increased in the northern region in recent years, suggesting changes in the geographic distribution of JE. Circular spatial scan statistic identified four significant spatio-temporal hotspots. Hotspots 1 and 4 were located in Shaannan and Guanzhong, which have fertile plains with ideal habitats for the development of *Cx. Tritaeniorhynchus*, and agree with the time duration of the outbreak in 2006. Hotspots 2 and 3 were located in Guanzhong and Shaanbei, respectively. Therefore, targeted measures such as disease and reservoir surveillance, estimation of seroprevalence, diagnosis, healthcare service training, and health education should be enhanced in these hotspots and in the northern region.

Several studies found strong associations between JE transmission and climate variations (14, 29–32). Climatic factors can change the living and proliferation environment of mosquitoes and lead to temporal and spatial changes in mosquito density (31, 32). The multivariable model indicated that temperature was a significant risk factor for JE. Temperature has multiple effects by affecting mosquito development and increasing viral replication in an amplifying host and vector (33). Global warming has significantly contributed to the increase in the geographic and seasonal distribution of vectors, including mosquitoes (34). We hypothesized that temperature was the most important environmental factor and, along with global warming, helped spread JE northward in the Shaanxi Province. In addition, relative humidity is known to be positively correlated with JE transmission, and temperature and relative humidity directly affect the distribution and population dynamics of mosquitoes (35, 36). Suitable relative humidity allows mosquitoes to survive longer and disperse further and directly affects water evaporation rates in mosquito-breeding sites, which affects the risk of pathogen transmission (14). A previous study reported that wind velocity affected mosquito oviposition (37), and high wind velocity may help mosquitoes disperse further. ENSO was a risk factor in our study. ENSO is a multi-year climate driver of local temperature and rainfall worldwide. It may form the biological basis for the relationship between ENSO and the incidence of JE (38).

Land use cover may be indirectly related to the prevalence of JE by impacting breeding sites, adult mosquito survival, and human activities (17, 39). Urban areas was negatively associated with the occurrence of JE. The sanitary conditions of urban environments are better, and urban dwellers have stronger self-protection awareness and behaviors, which likely reduce the likelihood of JE transmission comparing with rural areas, where are ideal for the development of *Cx. Tritaeniorhynchus*. Likewise, coniferous forest coverage was negatively correlated with disease incidence, which may be related to less human activity in these areas. However, few studies have evaluated the mechanism underlying this relationship, and these findings need to be further evaluated.

This study has several limitations. First, data on factors that could influence JEV distribution, such as personal health behavior, personal vaccination status, antibody positivity in the population, and mosquito population dynamics in administrative units, are not available or not applicable. Second, data on water bodies were extracted from the land cover map of ESA. *Cx. Tritaeniorhynchus* reproduces predominantly in clean or slightly polluted stagnant or slowly moving water, such as paddy fields, puddles, ditches, and reservoirs; however, only data from large water bodies such as rivers and lakes were included in the analysis. In addition, the method of analysis merit future well-designed to reveal the potential influencing factors of the transmission of JE. Notwithstanding, these findings provide the basis for proposing environmental changes and identifying areas where prevention and control efforts should be adopted.

In conclusion, our study elucidated the epidemic dynamics of human JE in Shaanxi and identified hotspots and the underlying risk factors for disease transmission. The results showed that there were changes in epidemic characteristics in the study area. The change of risk group highlights the increased need to formulate and implement targeted strategies and measures to reduce disease incidence in China. Further studies are needed to figure out whether this is also a northern trend of JE throughout China, not only in Shaanxi Province, so that China's CDC and CDC in other provinces can carry out timely and appropriate health policies and preventative measures to reduce the disease incidence.

## Data Availability Statement

The raw data supporting the conclusions of this article will be made available by the authors, without undue reservation.

## Ethics Statement

In this study, the ethics approval was not required for participation. The National Health Commission of China determined whether ethical approval was required for this study. In China, the collection of data from human Japanese encephalitis cases was part of routine public health surveillance, and such data collection was exempt from institutional review board assessment.

## Author Contributions

KL and ZS conceived the idea. KL, SS, and ZH collected the data. SS, HY, and ZY analyzed the data. SS, KL, and ZS wrote the manuscript. All authors contributed to manuscript revision, read, and approved the submitted version.

## Conflict of Interest

The authors declare that the research was conducted in the absence of any commercial or financial relationships that could be construed as a potential conflict of interest.

## Footnotes

^1^http://sxwjw.shaanxi.gov.cn/art/2009/10/9/art_362_50577.html

## Abbreviations

JE: Japanese Encephalitis
JEV: Japanese Encephalitis Virus
GIS: geographic information system
CDC: Center for Disease Control and Prevention
ENSO: El Niño-Southern Oscillation
RR: risk ratio
HR: hazard ratio
CI: confidence interval
EPI: Expanded Program on Immunization.
